# Changes of brain structure and structural covariance networks in Parkinson’s disease associated cognitive impairment

**DOI:** 10.3389/fnagi.2024.1449276

**Published:** 2024-09-26

**Authors:** Rong-Pei Liu, Guo-Liang Lin, Lu-Lu Ma, Shi-Shi Huang, Cheng-Xiang Yuan, Shi-Guo Zhu, Mei-Ling Sheng, Ming Zou, Jian-Hong Zhu, Xiong Zhang, Jian-Yong Wang

**Affiliations:** ^1^Department of Neurology, Institute of Geriatric Neurology, The Second Affiliated Hospital and Yuying Children’s Hospital, Wenzhou Medical University, Wenzhou, Zhejiang, China; ^2^Department of Preventive Medicine, Institute of Nutrition and Diseases, Wenzhou Medical University, Wenzhou, Zhejiang, China

**Keywords:** Parkinson’s disease, cognitive impairment, brain structure, network, magnetic resonance imaging

## Abstract

**Background:**

Cognitive impairment (CI) is common in Parkinson’s disease (PD). Multiple brain regions and their interactions are involved in PD associated CI. Magnetic resonance imaging (MRI) technology is a non-invasive method in investigating brain structure and inter-regional connections. In this study, by comparing cortical thickness, subcortical volume, and brain network topology properties in PD patients with and without CI, we aimed to understand the changes of brain structure and structural covariance network properties in PD associated CI.

**Methods:**

A total of 18 PD patients with CI and 33 PD patients without CI were recruited. Movement Disorder Society Unified Parkinson’s Disease Rating Scale, Hoehn and Yahr stage, Mini Mental State Examination Scale, Non-motor Symptom Rating Scale, Hamilton Anxiety Scale, and Hamilton Depression Scale were assessed. All participants underwent structural 3T MRI. Cortical thickness, subcortical volume, global and nodal network topology properties were measured.

**Results:**

Compared with PD patients without CI, the volumes of white matter, thalamus and hippocampus were lower in PD patients with CI. And decreased whole-brain local efficiency is associated with CI in PD patients. While the cortical thickness and nodal network topology properties were comparable between PD patients with and without CI.

**Conclusion:**

Our findings support the alterations of brain structure and disruption of structural covariance network are involved in PD associated CI, providing a new insight into the association between graph properties and PD associated CI.

## Introduction

Parkinson’s disease (PD) is a common neurodegenerative disorder characterized by motor symptoms bradykinesia, rest tremor, rigidity and postural instability (Postuma et al., 2015). With the progression of the disease, the prevalence of cognitive impairment (CI) in PD patients can reach as high as 83%, which greatly affects the quality of life of PD patients (Hely et al., 2008).

Different from Alzheimer’s disease (AD), CI in PD patients mainly involves executive and visuospatial functions (Aarsland et al., 2021). The mechanisms underlying CI in PD remain largely unclear, neurotransmitters disturbance in multiple brain regions, as well as abnormal deposition of substances such as α-synuclein and β-amyloid are believed to be involved (Aarsland et al., 2021). Difficulties in obtaining brain tissue from patients are a major obstacle to the progress of related research.

As a non-invasive method, magnetic resonance imaging (MRI) technology is widely used to explore changes in brain structure and function. In addition to conventional MRI, more advanced methods such as resting state functional MRI (rs-fMRI) and diffusion tensor imaging (DTI) adept at exploring the structural and functional connections between brain regions, and have revealed relevant characteristic changes in a variety of neurodegenerative diseases (Filippi et al., 2021; Firbank et al., 2016; Dopper et al., 2014). However, we have to admit that individual functional magnetic characteristics vary in different cognitive activity states (Cole et al., 2021).

Some analysis techniques based on brain MRI morphology, which are less sensitive to brain activity state (Bernhardt et al., 2010), complement rs-fMRI and DTI in investigating brain structure and inter-regional connections. These morphological features include cortical thickness, subcortical volume, whole-brain and nodal networks. Some previous studies using voxel-based morphometry method have explored gray matter (GM) changes in PD patients with CI. For instance, a study compared GM volumes of 12 PD patients with dementia and 12 PD patients without dementia, and found that patients with PD dementia had decreased bilateral superior temporal gyrus, bilateral posterior cingulate, left cingulate gyrus, right parahippocampal gyrus, right hippocampus, right precuneus, right cuneus, left inferior frontal gyrus and left insular lobe (Xia et al., 2013). Another study including 25 PD patients with mild CI (MCI) and 65 PD patients without CI found that PD with MCI is associated reduced thalamus and nucleus accumbens volumes (Mak et al., 2014). Their results were inconclusive, and they did not simultaneously calculate the white matter volume and network properties changes.

In this study, we measured cortical thickness, subcortical volumes, and brain network topology properties in a cohort of PD patients with and without CI, and aimed to understand the changes in brain structure and structural covariance network properties associated with CI in PD patients.

## Materials and methods

### Patients

A total of 51 PD patients were recruited from Department of Neurology, the Second Affiliated Hospital and Yuying Children’s Hospital from July 2019 to October 2021. All patients were diagnosed independently by two movement disorder neurologists according to the movement disorder society clinical diagnostic criteria for PD (Postuma et al., 2015). Exclusion criteria were (1) with history of other neurological or psychiatric disorders besides PD; (2) with history of alcohol or drug abuse; (3) with family history of PD or dementia; (4) with intracranial lesions detected by MRI. This study was approved by the ethics committee of the Second Affiliated Hospital and Yuying Children’s Hospital, Wenzhou Medical University. All participants signed written informed consents.

### Clinical evaluations and subtypes

Clinical data including age, gender, disease duration, years of education, Movement Disorder Society Unified Parkinson’s Disease Rating Scale (MDS-UPDRS), Hoehn and Yahr stage, Mini Mental State Examination Scale (MMSE), Non-motor Symptom Rating Scale (NMSS), Hamilton Anxiety Scale (HAMA), and Hamilton Depression Scale (HAMD) were assessed by face-to-face interview and physical examinations during the “OFF” state. All the patients were divided into PD with CI (including MCI and dementia) and PD without CI subtypes based on the MMSE score and their education level. In brief, subjects with MMSE score ≤ 19 for illiterate, ≤ 22 for elementary education and ≤ 26 for secondary education were considered with CI as previously described (Maimaitituerxun et al., 2023).

### MRI acquisition and preprocessing

All participants were scanned on a 3 Tesla GE-Discovery 750 scanner, and T1-anatomical brain images were acquired with the parameters described before (Yang et al., 2021). After converting to NIFTI format, images were analyzed using FreeSurfer version 7.3.1^1^ (Dale et al., 1999; Fischl et al., 1999). The size of a Gaussian smoothing kernel was 10 mm, which was adopted in previous studies (Gerrits et al., 2016; Tessitore et al., 2016). FreeSurfer generated a surface model for the cortex, and automatically parcellated the cortex into 31 regions-of-interest (ROIs) per hemisphere, according to the Desikan-Killiany-Tourville protocol. For each of the 62 ROIs, FreeSurfer calculated the average cortical thickness. To ensure the quality of T1 images, FreeView visualization tool was utilized for visual inspection.

Subcortical volumes including thalamus, caudate, putamen, pallidum, hippocampus, amygdala, accumbens-area, total white matter (WM), total GM, white matter hyperintensity (WMH), third ventricle (3rd-Ventricle), fourth ventricle (4th-Ventricle), and lateral ventricle were calculated automatically with FreeSurfer’s procedure for volumetric measures, including skull stripping, affine registration with MNI305 space, volumetric labeling, variation correction and visualizing volume (Fischl et al., 2002). During the processing pipeline, FreeSurfer checked the quality of segmentations and generated a quality report, which ensured the validity of the data.

### Network construction and graph theory analysis

The 76 morphological features (62 cortical thicknesses and 14 subcortical GM volumes) were adjusted with age, gender, and estimated total intracranial volume (eTIV), as previous described (Yun et al., 2020). The resulting residuals were z-score transformed by using the mean and standard deviation values of each ROIs calculated from PD without CI group. The similarity between the ROIs represented the edge of the network, which was calculated using the following formula: 1/*e*^[(*zscoreofxthROI*-*zscoreofythROI*)^2^]^ (Yun et al., 2020). The networks were thresholded (using a network density of 0.01 to 0.5, with interval of 0.01) and binarized. According to the following criteria: (1) more than 80% of are connected to other nodes; (2) modularity > 0.3; (3) small-world > 1, the network density range was finally determined as 0.1–0.34, with an interval of 0.01.

From these thresholded and binarized networks, six global metrics were determined: small-world index (σ), normalized characteristic path length (λ), normalized clustering coefficient (γ), assortativity, global efficiency (Eglob), and local efficiency (Eloc). The nodal network topology properties include γ, Eloc, degree centrality, and betweenness centrality (Watts and Strogatz, 1998). Estimation of the global and nodal network topology properties was done using the Graph Theoretical Network Analysis (GRETNA)^2^ package in MATLAB R2022a.

### Statistical analysis

The data were analyzed using Rstudio 2022.12.0. The normal distribution of the data was assessed by Shapiro–Wilk test. Differences in MDS-UPDRS Total, MDS-UPDRS I, MMSE and eTIV between PD with CI and PD without CI groups were analyzed by unpaired two-tailed *t*-test. Differences in age, disease duration, years of education, MDS-UPDRS II, MDS-UPDRS III, Hoehn and Yahr stage, NMSS, HAMA and HAMD were analyzed by Mann–Whitney test. Difference in gender distribution was assessed by Chi square test. Multivariate analysis was performed by binary logistic regression with age, gender and disease duration as covariates. The differences in cortical thickness were analyzed in FreeSurfer’s statistical program QDEC 1.5 (Gerrits et al., 2016). Global and nodal network topology properties were compared using GRETNA, and false discovery rate (FDR) was used to correct the multiple comparisons. A two-tailed *P* < 0.05 was considered statistically significant.

## Results

### Demographic and clinical characteristics of PD patients

There were 18 cases in PD with CI group and 33 cases in PD without CI group. The demographic and clinical characteristics were summarized in Table 1. Age, gender, disease duration, years of education, MDS-UPDRS score, MDS-UPDRS I score, MDS-UPDRS II score, MDS-UPDRS III score, Hoehn and Yahr stage, NMSS score, HAMA score, HAMD score and eTIV were comparable between the two groups (*P* > 0.05; Table 1). While MMSE score was significantly lower in PD with CI group (*P* < 0.001; Table 1).

**TABLE 1:** Demographic and clinical characteristics of PD patients.

	PD with CI	PD without CI	*P*	*P* *
Subject, *n* (%)	18 (35.3)	33 (64.7)	–	–
Age, years (IR)	70.50 (67.25–71.75)	68.00 (66.00–71.00)	0.363^a^	–
Gender, F/M	8/10	20/13	0.416^b^	–
Duration, years (IR)	3.00 (2.00–5.00)	4.00 (1.50–5.00)	0.705^a^	–
Education, years (IR)	6.00 (0.00–6.00)	6.00 (0.00–6.00)	0.827^a^	–
MDS-UPDRS total, mean ± SD	53.30 ± 38.50	54.20 ± 29.50	0.927^c^	0.714^d^
MDS-UPDRS I, mean ± SD	10.39 ± 8.26	10.76 ± 5.25	0.866^c^	0.594^d^
MDS-UPDRS II, (IR)	8.50 (3.50–16.75)	12.00 (9.00–16.00)	0.225^a^	0.332^d^
MDS-UPDRS III, (IR)	24.50 (14.50–41.75)	20.00 (17.00–39.00)	0.984^a^	0.999^d^
Hoehn and Yahr, (IR)	2.00 (2.00–2.75)	2.00 (2.00–2.50)	0.864^a^	0.664^d^
MMSE, mean ± SD	18.80 ± 4.20	25.90 ± 2.70	< 0.001^c^	0.007^d^
NMSS, (IR)	51.50 (16.75–92.25)	27.00 (19.75–47.25)	0.323^a^	0.265^d^
HAMA, (IR)	10.00 (7.50–20.25)	7.00 (4.00–13.00)	0.204^a^	0.119^d^
HAMD, (IR)	10.50 (6.25–17.25)	9.00 (3.00–14.00)	0.212^a^	0.070^d^
eTIV, mm^3^, mean ± SD	1,518,817.00 ± 255,734.70	1,387,449.00 ± 153,136.70	0.058^c^	0.055^d^

CI, cognitive impairment; eTIV, estimated total intracranial volume; F, female; HAMA: Hamilton Anxiety Scale; HAMD, Hamilton Depression Scale; IR, interquartile range; M, male; MDS-UPDRS, Movement Disorder Society-Unified Parkinson’s Disease Rating Scale; MMSE, Mini Mental State Examination Scale; NMSS, Non-motor Symptom Rating Scale; PD, Parkinson’s disease; SD, standard deviation. *Adjusted with age, gender and disease duration. ^a^Analyzed by Mann–Whitney test. ^b^Analyzed by Chi square test. ^c^Analyzed by unpaired two-tailed *t*-test. ^d^Analyzed by binary logistic regression.

### Subcortical volume and cortical thickness in PD patients with and without CI

**FIGURE 1:**
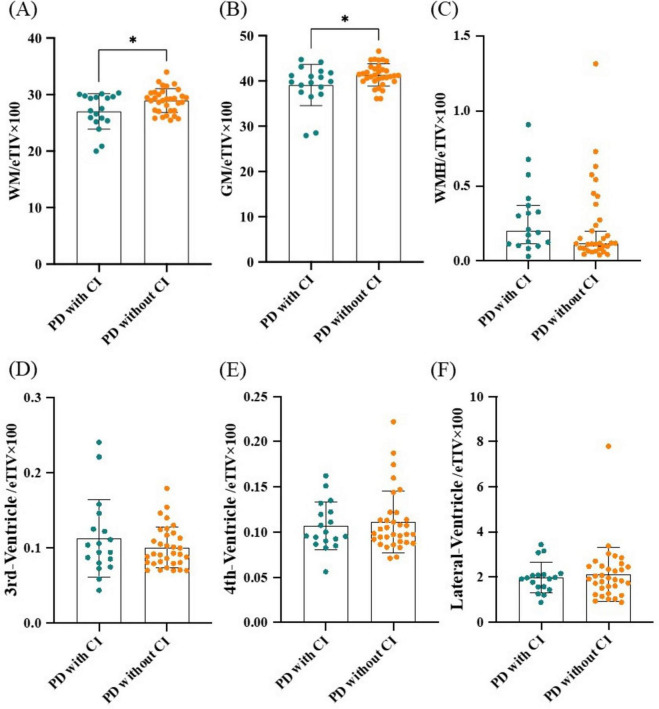
Comparison of subcortical volumes between PD patients with and without CI. **(A)** WM/eTIV. **(B)** GM/eTIV. **(C)** WMH/eTIV. **(D)** 3rd-Ventricle/eTIV. **(E)** 4th-Ventricle/eTIV. **(F)** Lateral-Ventricle/eTIV. *n* = 18 for PD patients with CI; *n* = 33 for PD patients without CI. Values are expressed as means ± standard error. *P < 0.05. CI, Cognitive impairment; eTIV, estimated total intracranial volume; GM, Gray matter; PD, Parkinson’s disease; WM, White matter; WMH, White matter hyperintensity.

Subcortical volume including WM, GM, WMH, 3rd-ventricle, 4th-ventricle and lateral-ventricle were calculated and their ratios to eTIV were compared between PD patients with and without CI. Results showed that WM/eTIV and GM/eTIV were significantly lower in PD with CI group than in PD without CI group (*P* = 0.012 and *P* = 0.026, respectively; Figures 1A, B). No significant difference was found in WMH/eTIV, 3rd-ventricle/eTIV, 4th-ventricle/eTIV and lateral-ventricle/eTIV between the two groups (Figures 1C–F). Further binary logistic regression analysis adjusted for age, gender, and disease duration confirmed a decrease in WM/eTIV in PD patients with CI (*P* = 0.012, OR 0.72, 95% confidence interval 0.50–0.95; Table 2).

**TABLE 2:** Multivariate risk analysis for PD with CI.

Factors	B	*P* ^a^	OR	95% confidence interval	
				Lower	Upper
WM/eTIV	−0.32	0.041	0.72	0.50	0.95
GM/eTIV	−0.21	0.080	0.81	0.61	1.00
WMH/eTIV	0.72	0.540	2.06	0.17	22.50
3rd-Ventricle/eTIV	9.60	0.304	1.48 × 10^4^	0.15 × 10^–2^	3.33 × 10^12^
4th-Ventricle/eTIV	−5.18	0.622	0.56 × 10^–2^	5.78 × 10^–13^	2.17 × 10^6^
Lateral-Ventricle/eTIV	−0.23	0.578	0.80	0.29	1.53

CI, cognitive impairment; eTIV, estimated total intracranial volume; GM, gray matter; OR, odds ratio; PD, Parkinson’s disease; WM, white matter; WMH, white matter hyperintensity. ^a^Adjusted with age, gender and disease duration.

**FIGURE 2:**
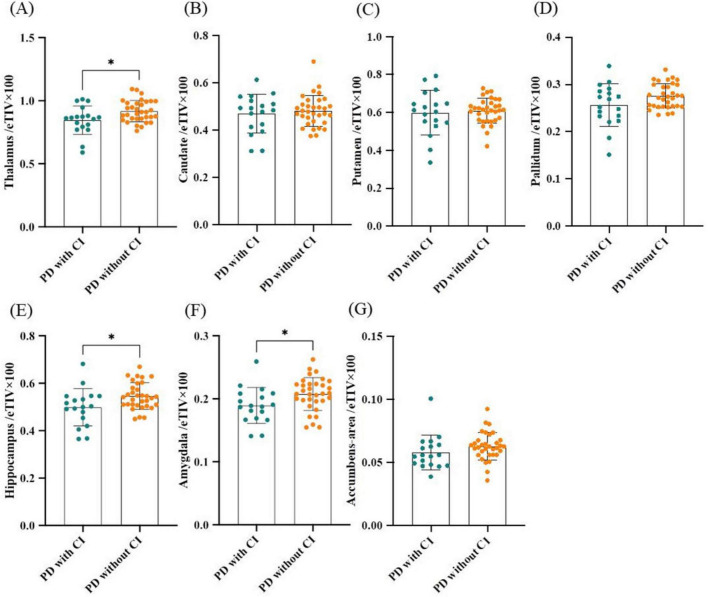
Comparison of subcortical gray matter volumes between PD patients with and without CI. **(A)** Thalamus/eTIV. **(B)** Caudate/eTIV. **(C)** Putamen/eTIV. **(D)** Pallidum/eTIV. **(E)** Hippocampus/eTIV. **(F)** Amygdala/eTIV. **(G)** Accumbens-area/eTIV. *n* = 18 for PD patients with CI; *n* = 33 for PD patients without CI. Values are expressed as means ± standard error. *P < 0.05. CI, Cognitive impairment; eTIV, estimated total intracranial volume; PD, Parkinson’s disease.

Next, subcortical GM volume/eTIV including thalamus/eTIV, caudate/eTIV, putamen/eTIV, pallidum/eTIV, hippocampus/eTIV, amygdala/eTIV and accumbens-area/eTIV were compared between PD with and without CI groups. Results showed that thalamus/eTIV, hippocampus/eTIV and amygdala/eTIV were significantly lower in PD patients with CI (*P* = 0.013, *P* = 0.016, and *P* = 0.025; Figures 2A, E, F). The decline in thalamus/eTIV and hippocampus/eTIV was further confirmed by binary logistic regression analysis adjusted for age, gender, and disease duration (*P* = 0.046, OR 1.10 × 10^–4^, 95% confidence interval 3.03 × 10^–9^ to 3.08 × 10^–1^ and *P* = 0.044, OR 4.30 × 10^–6^, 95% confidence interval 5.69 × 10^–12^ to 2.81 × 10^–1^, respectively; Table 3). No significant difference was found in caudate/eTIV, putamen/eTIV, pallidum/eTIV and accumbens-area/eTIV between the two groups (Figure 2 and Table 3).

**TABLE 3:** Multivariate risk analysis for PD with CI.

Factors	B	*P* ^a^	OR	95% confidence interval	
				Lower	Upper
Thalamus/eTIV	−9.11	0.046	1.10 × 10^–4^	3.03 × 10^–9^	3.08 × 10^–1^
Caudate/eTIV	−1.03	0.826	0.36	2.85 × 10^–5^	3,505.18
Putamen/eTIV	−1.03	0.769	0.36	0.31 × 10^–2^	409.76
Pallidum/eTIV	−16.43	0.102	7.34 × 10^–8^	2.85 × 10^–17^	8.65
Hippocampus/eTIV	−12.36	0.044	4.30 × 10^–6^	5.69 × 10^–12^	2.81 × 10^–1^
Amygdala/eTIV	−25.03	0.064	1.35 × 10^–11^	2.18 × 10^–24^	1.03
Accumbens-area/eTIV	−35.95	0.232	2.44 × 10^–16^	2.59 × 10^–44^	3.51 × 10^8^

CI, cognitive impairment; eTIV, estimated total intracranial volume; OR, odds ratio; PD, Parkinson’s disease. ^a^Adjusted with age, gender and disease duration.

The cortical thickness was comparable between PD patients with and without CI (Supplementary Table 1).

### Whole-brain and nodal network topology properties in PD patients with and without CI

Whole-brain network topology properties including σ, λ, γ, assortativity, Eglob, Eloc, and nodal network topology properties including γ, Eloc, degree centrality, betweenness centrality were calculated and analyzed in PD patients with and without CI. Results showed that whole-brain Eloc in PD with CI was significantly decreased (Table 4). No significant differences were found in the remaining whole-brain topology properties between the two groups (Table 4), none was found in nodal network topology properties (Supplementary Tables 2–5).

**TABLE 4:** Whole-brain network topology properties of PD patients with and without CI.

Properties	PD with CI (*n* = 18)	PD without CI (*n* = 33)	*t*	*P* *
σ^a^, mean ± SD	0.469 ± 0.070	0.494 ± 0.055	−1.450	0.154
λ^a^, mean ± SD	0.340 ± 0.010	0.341 ± 0.011	−0.354	0.725
γ^a^, mean ± SD	0.679 ± 0.091	0.719 ± 0.076	−1.685	0.098
Assortativity^a^, mean ± SD	0.182 ± 0.011	0.179 ± 0.015	0.781	0.438
Eglob^a^, mean ± SD	0.101 ± 0.004	0.102 ± 0.002	−1.120	0.268
Eloc^a^, mean ± SD	0.201 ± 0.006	0.204 ± 0.003	−2.139	0.037

σ, small-world index; λ, normalized characteristic path length; γ, normalized clustering coefficient; CI, cognitive impairment; Eglob, global efficiency; Eloc, local efficiency; FDR, false discovery rate; PD, Parkinson’s disease; SD, standard deviation. *Adjusted by FDR correction. ^a^All values are expressed as the area under the curve across the density range and are adjusted with age and gender.

## Discussion

CI is one of the most common non-motor symptoms of PD patients, and it is believed to be associated with abnormalities in the structural and functional connectivity of multiple brain regions. In the current study, we analyzed cortical thickness, subcortical volume, and brain network topology properties in 18 PD patients with CI and 33 PD patients without CI. Our results demonstrate that WM, thalamus and hippocampus volumes are lower in PD patients with CI than in those without CI. And decreased whole-brain Eloc is associated with CI in PD patients. We did not find differences in cortical thickness and nodal network topology properties between PD patients with and without CI.

In recent years, the non-motor symptoms of PD have received increasing attention (Kalia and Lang, 2015). As the disease progresses, CI in PD patients seems inevitable, which greatly affects their quality of life (Hely et al., 2008; Aarsland et al., 2005). It is believed that PD associated CI involves disturbances in multiple brain regions and neurotransmitter systems (Aarsland et al., 2021).

Some neurocognitive disorders, such as AD and frontotemporal dementia, are more likely to involve the cerebral cortex and cause corresponding symptoms. Therefore, in this study, we first measured the cortical thickness. However, we did not find any difference in cortical thickness between PD patients with CI and those without CI. Similarly, a previous study compared cortical thickness between 29 PD dementia patients and 76 cognitively normal controls, and found that regional cortical thickness was indistinguishable between the two groups (Colloby et al., 2020). However, some other studies have reported that PD associated CI is associated with cortical atrophy in frontal lobe (Gao et al., 2017), left precuneus (Pereira et al., 2014), parietotemporal lobe (Segura et al., 2014), right temporal lobe, right insula, right inferior frontal gyrus and right supplementary motor area (Hanganu et al., 2014). The diversity of results may arise from differences in study designs, cognitive status, ethnicity, and disease duration. For example, only one study was a cohort study that focused on the rate of cortical thinning in different groups (Hanganu et al., 2014), while several other cross-sectional studies focused on baseline differences in cortical thickness between groups (Colloby et al., 2020; Gao et al., 2017; Pereira et al., 2014; Segura et al., 2014). On the other hand, one study compared the differences in cortical thickness between patients with PD dementia and healthy controls using samples from different studies, whereas other studies included comparisons between PD-MCI and PD without MCI groups (Gao et al., 2017; Pereira et al., 2014; Segura et al., 2014; Hanganu et al., 2014). What’s more, the populations in these different studies across China (Gao et al., 2017), Spain (Segura et al., 2014), Canada (Hanganu et al., 2014) and Parkinson’s Progression Markers Initiative (Pereira et al., 2014). In one study, there was a significant difference in disease duration between PD-MCI and PD without MCI groups, which may also interfere with the analysis of results (Segura et al., 2014). In order to understand the relationship between cortical thickness and CI in PD more accurately, future investigations are warranted in larger populations.

Next, subcortical volumes including WM, GM, WMH and ventricles were compared between PD patients with and without CI. Our results showed that WM volume in PD patients with CI is lower than in PD patients without CI. WM is composed of nerve fibers and plays a major role in coordinating communication between brain regions, the dysfunction of which is associated with cognitive slowing, executive dysfunction and memory retrieval deficit (Fields, 2008; Filley and Fields, 2016). It has been noted that changes in WM microstructure precede those in GM in PD patients (Andica et al., 2019). Previous studies have linked WM lesions and hyperintensities to PD associated CI (Melzer et al., 2013; Hattori et al., 2012; Carvalho de Abreu et al., 2023). Our study further indicates that the reduction of WM volume is associated with CI in PD patients. Based on this finding, we suggest that sufficient attention should be paid to WM-related memory and executive functions in PD patients, so that CI can be detected and intervened early.

Subcortical GM is a group of scattered GM islets beneath cerebral cortex. They have their own unique functions and also play a role in integrating nerve impulses from different brain regions. In this study, we found that PD patients with CI had smaller thalamus and hippocampus volumes compared with PD patients without CI. Several previous studies have explored the relationship between subcortical GM change and PD associated CI. Interestingly, reduced hippocampus (Chung et al., 2017; Foo et al., 2017; Schneider et al., 2017) and thalamus (Mak et al., 2014; Chung et al., 2017; Foo et al., 2017) volumes in PD patients with CI seem to be a consistent finding across studies. In addition, right caudate (Chung et al., 2017), caudate, presubiculum, cornu ammonis (Foo et al., 2017), and nucleus accumbens (Mak et al., 2014) have also been reported to be associated with CI in PD. However, they have not yet been replicated in different studies. Therefore, we believe that learning, memory, and sensory integration mediated by the hippocampus and thalamus are worthy of attention when studying PD associated CI.

The application of brain network topological properties is based on the assumption that neurons in interconnected regions share similar developmental and maturational influences, so they have covariant morphological properties (Alexander-Bloch et al., 2013). In the current study, we found whole-brain Eloc is decreased in PD patients with CI. A previous study analyzed the global and regional network topology properties of 33 PD patients with mild CI (MCI) and 90 cognitively normal PD patients from Parkinson’s Progression Markers Initiative, and found that PD patients with MCI showed reduced global average correlation strength and lower regional efficiency in frontal and parietal regions compared with cognitively normal PD patients (Pereira et al., 2015). It is worth noting that unlike our study, which constructed a network for each individual, this study constructed a network for each group. Here, we insist that PD patients with CI are disrupted in the coordination of the brain network.

The interpretation of topology properties changes is difficult, because the precise biological interpretation of each topology property remains unclear. Considering a topological property as a characteristic signature of any specific disease at a single time point may lead to ambiguous conclusions (Douw et al., 2019). However, previous study has shown that topological properties can monitor disease progression from more perspectives (Tijms et al., 2013). Longitudinal studies are needed to further validate the value of whole-brain Eloc in monitoring the progression of PD associated CI.

Meanwhile, we have to acknowledge certain limitations. Due to the limited number of cases, we did not stratify patients according to different cognitive levels. Meanwhile, our study only included a global efficiency scale and no tool to measure daily life functioning. Therefore, the division between patients with and without CI didn’t follow the published guidelines for PD-MCI (Litvan et al., 2012) and PD dementia (Emre et al., 2007). In addition, our study lacks longitudinal observation, the presence of which would make the conclusions more accurate and valuable.

In summary, our study demonstrates a reduction of WM, thalamus and hippocampus volumes and a decrease of whole-brain Eloc in PD patients with CI. Our findings support the alteration of brain structure and disruption of structural covariance network are involved in PD associated CI.

## Data Availability

The original contributions presented in this study are included in the article/Supplementary material, further inquiries can be directed to the corresponding authors.

## Footnotes

1
http://surfer.nmr.mgh.harvard.edu

2
www.nitrc.org/projects/gretna/
