# Supplementary material for: Changes of brain structure and structural covariance networks in Parkinson’s disease associated cognitive impairment
Source: Front Aging Neurosci. 2024 Sep 26;16:1449276. doi: 10.3389/fnagi.2024.1449276 (PMC11464354; doi:10.3389/fnagi.2024.1449276)
Supplement: Supplementary file 1 [file Table_1.DOCX]

Supplementary table 1. Cortical thickness of PD patients with and without CI

|  | PD with CI (n = 18) | PD without CI (n = 33) | *P* value after FDR correction^*^ |
| --- | --- | --- | --- |
| Caudal anterior cingulate-L | 2.47 ± 0.16 | 2.45 ± 0.16 | 0.929 |
| Caudal middle frontal-L | 2.47 ± 0.13 | 2.50 ± 0.12 | 0.929 |
| Cuneus-L | 1.93 ± 0.12 | 1.94 ± 0.09 | 0.929 |
| Entorhinal-L | 3.13 ± 0.37 | 3.17 ± 0.30 | 0.929 |
| Fusiform-L | 2.40 ± 0.14 | 2.45 ± 0.09 | 0.929 |
| Inferior parietal-L | 2.21 ± 0.09 | 2.25 ± 0.08 | 0.929 |
| Inferior temporal-L | 2.65 ± 0.17 | 2.63 ± 0.13 | 0.929 |
| Isthmus cingulate-L | 2.10 ± 0.16 | 2.15 ± 0.17 | 0.929 |
| Lateral occipital-L | 2.01 ± 0.10 | 2.01 ± 0.09 | 0.945 |
| Lateral orbitofrontal-L | 2.64 ± 0.20 | 2.62 ± 0.14 | 0.929 |
| Lingual-L | 1.99 ± 0.12 | 2.01 ± 0.10 | 0.929 |
| Medial orbitofrontal-L | 2.47 ± 0.19 | 2.42 ± 0.14 | 0.929 |
| Middle temporal-L | 2.53 ± 0.14 | 2.54 ± 0.11 | 0.982 |
| Parahippocampal-L | 2.42 ± 0.21 | 2.42 ± 0.24 | 0.929 |
| Paracentral-L | 2.40 ± 0.14 | 2.44 ± 0.13 | 0.929 |
| Pars opercularis-L | 2.50 ± 0.11 | 2.50 ± 0.13 | 0.989 |
| Pars orbitalis-L | 2.61 ± 0.13 | 2.60 ± 0.14 | 0.929 |
| Pars triangularis-L | 2.41 ± 0.14 | 2.39 ± 0.13 | 0.929 |
| Pericalcarine-L | 1.87 ± 0.16 | 1.86 ± 0.13 | 0.952 |
| Postcentral-L | 2.05 ± 0.09 | 2.07 ± 0.10 | 0.929 |
| Posterior cingulate-L | 2.32 ± 0.14 | 2.28 ± 0.12 | 0.929 |
| Precentral-L | 2.48 ± 0.08 | 2.50 ± 0.11 | 0.929 |
| Precuneus-L | 2.24 ± 0.11 | 2.25 ± 0.08 | 0.996 |
| Rostral anterior cingulate-L | 2.63 ± 0.15 | 2.60 ± 0.13 | 0.929 |
| Rostral middle frontal-L | 2.37 ± 0.14 | 2.39 ± 0.11 | 0.929 |
| Superior frontal-L | 2.62 ± 0.14 | 2.62 ± 0.11 | 0.929 |
| Superior parietal-L | 2.11 ± 0.09 | 2.13 ± 0.08 | 0.929 |
| Superior temporal-L | 2.60 ± 0.10 | 2.63 ± 0.11 | 0.929 |
| Supramarginal-L | 2.33 ± 0.11 | 2.35 ± 0.09 | 0.929 |
| Transverse temporal-L | 2.24 ± 0.14 | 2.30 ± 0.18 | 0.929 |
| Insula-L | 2.84 ± 0.16 | 2.86 ± 0.14 | 0.929 |
| Caudal anterior cingulate-R | 2.39 ± 0.20 | 2.33 ± 0.18 | 0.929 |
| Caudal middle frontal-R | 2.44 ± 0.13 | 2.44 ± 0.11 | 0.982 |
| Cuneus-R | 1.95 ± 0.11 | 1.92 ± 0.13 | 0.929 |
| Entorhinal-R | 3.28 ± 0.28 | 3.20 ± 0.35 | 0.929 |
| Fusiform-R | 2.58 ± 0.14 | 2.57 ± 0.11 | 0.929 |
| Inferior parietal-R | 2.26 ± 0.10 | 2.29 ± 0.11 | 0.929 |
| Inferior temporal-R | 2.58 ± 0.15 | 2.58 ± 0.13 | 0.929 |
| Isthmus cingulate-R | 2.16 ± 0.16 | 2.18 ± 0.16 | 0.952 |
| Lateral occipital-R | 2.09 ± 0.13 | 2.10 ± 0.10 | 0.938 |
| Lateral orbitofrontal-R | 2.56 ± 0.18 | 2.54 ± 0.14 | 0.929 |
| Lingual-R | 2.01 ± 0.16 | 2.00 ± 0.13 | 0.929 |
| Medial orbitofrontal-R | 2.47 ± 0.18 | 2.42 ± 0.11 | 0.929 |
| Middle temporal-R | 2.52 ± 0.11 | 2.57 ± 0.11 | 0.929 |
| Parahippocampal-R | 2.45 ± 0.15 | 2.47 ± 0.20 | 0.982 |
| Paracentral-R | 2.43 ± 0.17 | 2.46 ± 0.14 | 0.929 |
| Pars opercularis-R | 2.44 ± 0.12 | 2.41 ± 0.10 | 0.929 |
| Pars orbitalis-R | 2.45 ± 0.13 | 2.52 ± 0.16 | 0.929 |
| Pars triangularis-R | 2.37 ± 0.14 | 2.30 ± 0.11 | 0.929 |
| Pericalcarine-R | 1.79 ± 0.17 | 1.83 ± 0.17 | 0.929 |
| Postcentral-R | 2.05 ± 0.12 | 2.06 ± 0.12 | 0.929 |
| Posterior cingulate-R | 2.33 ± 0.10 | 2.32 ± 0.13 | 0.929 |
| Precentral-R | 2.39 ± 0.18 | 2.43 ± 0.15 | 0.929 |
| Precuneus-R | 2.29 ± 0.11 | 2.31 ± 0.09 | 0.929 |
| Rostral anterior cingulate-R | 2.67 ± 0.17 | 2.61 ± 0.15 | 0.929 |
| Rostral middle frontal-R | 2.31 ± 0.10 | 2.31 ± 0.09 | 0.929 |
| Superior frontal-R | 2.54 ± 0.12 | 2.57 ± 0.13 | 0.929 |
| Superior parietal-R | 2.16 ± 0.12 | 2.18 ± 0.11 | 0.929 |
| Superior temporal-R | 2.59 ± 0.13 | 2.62 ± 0.11 | 0.929 |
| Supramarginal-R | 2.31 ± 0.13 | 2.35 ± 0.09 | 0.929 |
| Transverse temporal-R | 2.30 ± 0.18 | 2.37 ± 0.16 | 0.929 |
| Insula-R | 2.86 ± 0.15 | 2.81 ± 0.15 | 0.929 |

All values are expressed as mean ± SD. CI: Cognitive impairment; FDR: False discovery rate; L: Left; PD: Parkinson’s disease; R: Right; SD: Standard deviation

^*^ Adjusted with age, gender and disease duration

Supplementary table 2. Nodal clustering coefficient of PD patients with and without CI

|  | PD with CI (n = 18) | PD without CI (n = 33) | *P* value after FDR correction |
| --- | --- | --- | --- |
| Caudal anterior cingulate-L | 0.185 ± 0.020 | 0.177 ± 0.035 | 0.827 |
| Caudal middle frontal-L | 0.173 ± 0.030 | 0.179 ± 0.022 | 0.827 |
| Cuneus-L | 0.168 ± 0.035 | 0.180 ± 0.033 | 0.765 |
| Entorhinal-L | 0.160 ± 0.068 | 0.175 ± 0.036 | 0.779 |
| Fusiform-L | 0.161 ± 0.067 | 0.186 ± 0.018 | 0.765 |
| Inferior parietal-L | 0.189 ± 0.020 | 0.182 ± 0.020 | 0.765 |
| Inferior temporal-L | 0.176 ± 0.035 | 0.171 ± 0.048 | 0.895 |
| Isthmus cingulate-L | 0.189 ± 0.015 | 0.175 ± 0.036 | 0.765 |
| Lateral occipital-L | 0.181 ± 0.012 | 0.175 ± 0.036 | 0.833 |
| Lateral orbitofrontal-L | 0.170 ± 0.045 | 0.184 ± 0.019 | 0.765 |
| Lingual-L | 0.181 ± 0.015 | 0.176 ± 0.030 | 0.833 |
| Medial orbitofrontal-L | 0.174 ± 0.050 | 0.185 ± 0.014 | 0.765 |
| Middle temporal-L | 0.173 ± 0.014 | 0.182 ± 0.024 | 0.765 |
| Parahippocampal-L | 0.180 ± 0.014 | 0.174 ± 0.039 | 0.833 |
| Paracentral-L | 0.180 ± 0.034 | 0.185 ± 0.018 | 0.833 |
| Pars opercularis-L | 0.179 ± 0.011 | 0.180 ± 0.017 | 0.899 |
| Pars orbitalis-L | 0.179 ± 0.019 | 0.173 ± 0.040 | 0.833 |
| Pars triangularis-L | 0.183 ± 0.013 | 0.181 ± 0.029 | 0.895 |
| Pericalcarine-L | 0.185 ± 0.016 | 0.185 ± 0.019 | 0.974 |
| Postcentral-L | 0.183 ± 0.013 | 0.188 ± 0.019 | 0.779 |
| Posterior cingulate-L | 0.177 ± 0.044 | 0.187 ± 0.016 | 0.765 |
| Precentral-L | 0.182 ± 0.014 | 0.184 ± 0.016 | 0.833 |
| Precuneus-L | 0.180 ± 0.048 | 0.178 ± 0.030 | 0.937 |
| Rostral anterior cingulate-L | 0.186 ± 0.022 | 0.184 ± 0.020 | 0.895 |
| Rostral middle frontal-L | 0.188 ± 0.014 | 0.183 ± 0.017 | 0.779 |
| Superior frontal-L | 0.178 ± 0.018 | 0.180 ± 0.023 | 0.895 |
| Superior parietal-L | 0.188 ± 0.021 | 0.185 ± 0.016 | 0.833 |
| Superior temporal-L | 0.187 ± 0.011 | 0.175 ± 0.019 | 0.765 |
| Supramarginal-L | 0.175 ± 0.028 | 0.184 ± 0.015 | 0.765 |
| Transverse temporal-L | 0.181 ± 0.018 | 0.177 ± 0.037 | 0.859 |
| Insula-L | 0.177 ± 0.029 | 0.186 ± 0.023 | 0.765 |
| Thalamus-L | 0.173 ± 0.027 | 0.180 ± 0.033 | 0.833 |
| Caudate-L | 0.186 ± 0.016 | 0.168 ± 0.046 | 0.765 |
| Putamen-L | 0.145 ± 0.075 | 0.173 ± 0.043 | 0.765 |
| Pallidum-L | 0.155 ± 0.075 | 0.166 ± 0.054 | 0.833 |
| Hippocampus-L | 0.173 ± 0.045 | 0.178 ± 0.028 | 0.840 |
| Amygdala-L | 0.170 ± 0.042 | 0.173 ± 0.044 | 0.899 |
| Accumbens-area-L | 0.167 ± 0.054 | 0.175 ± 0.041 | 0.833 |
| Caudal anterior cingulate-R | 0.162 ± 0.061 | 0.167 ± 0.053 | 0.895 |
| Caudal middle frontal-R | 0.178 ± 0.027 | 0.186 ± 0.015 | 0.765 |
| Cuneus-R | 0.185 ± 0.015 | 0.186 ± 0.018 | 0.895 |
| Entorhinal-R | 0.174 ± 0.048 | 0.177 ± 0.027 | 0.895 |
| Fusiform-R | 0.166 ± 0.052 | 0.179 ± 0.035 | 0.779 |
| Inferior parietal-R | 0.190 ± 0.019 | 0.185 ± 0.013 | 0.765 |
| Inferior temporal-R | 0.179 ± 0.021 | 0.182 ± 0.034 | 0.895 |
| Isthmus cingulate-R | 0.182 ± 0.013 | 0.181 ± 0.038 | 0.937 |
| Lateral occipital-R | 0.168 ± 0.057 | 0.177 ± 0.036 | 0.833 |
| Lateral orbitofrontal-R | 0.181 ± 0.015 | 0.185 ± 0.018 | 0.827 |
| Lingual-R | 0.189 ± 0.017 | 0.164 ± 0.054 | 0.765 |
| Medial orbitofrontal-R | 0.171 ± 0.047 | 0.180 ± 0.037 | 0.827 |
| Middle temporal-R | 0.193 ± 0.024 | 0.176 ± 0.032 | 0.765 |
| Parahippocampal-R | 0.181 ± 0.014 | 0.177 ± 0.029 | 0.833 |
| Paracentral-R | 0.178 ± 0.047 | 0.178 ± 0.036 | 0.999 |
| Pars opercularis-R | 0.179 ± 0.023 | 0.189 ± 0.019 | 0.765 |
| Pars orbitalis-R | 0.169 ± 0.041 | 0.172 ± 0.040 | 0.895 |
| Pars triangularis-R | 0.161 ± 0.051 | 0.179 ± 0.037 | 0.765 |
| Pericalcarine-R | 0.188 ± 0.015 | 0.177 ± 0.036 | 0.765 |
| Postcentral-R | 0.168 ± 0.045 | 0.182 ± 0.039 | 0.765 |
| Posterior cingulate-R | 0.172 ± 0.046 | 0.165 ± 0.045 | 0.840 |
| Precentral-R | 0.166 ± 0.051 | 0.171 ± 0.045 | 0.895 |
| Precuneus-R | 0.181 ± 0.012 | 0.186 ± 0.019 | 0.779 |
| Rostral anterior cingulate-R | 0.184 ± 0.016 | 0.173 ± 0.046 | 0.779 |
| Rostral middle frontal-R | 0.180 ± 0.021 | 0.186 ± 0.020 | 0.779 |
| Superior frontal-R | 0.185 ± 0.012 | 0.181 ± 0.018 | 0.827 |
| Superior parietal-R | 0.185 ± 0.022 | 0.190 ± 0.018 | 0.827 |
| Superior temporal-R | 0.177 ± 0.013 | 0.183 ± 0.016 | 0.765 |
| Supramarginal-R | 0.162 ± 0.06 | 0.183 ± 0.017 | 0.765 |
| Transverse temporal-R | 0.177 ± 0.029 | 0.179 ± 0.028 | 0.895 |
| Insula-R | 0.183 ± 0.012 | 0.184 ± 0.036 | 0.937 |
| Thalamus-R | 0.177 ± 0.052 | 0.177 ± 0.039 | 0.974 |
| Caudate-R | 0.179 ± 0.023 | 0.167 ± 0.052 | 0.827 |
| Putamen-R | 0.157 ± 0.069 | 0.179 ± 0.036 | 0.765 |
| Pallidum-R | 0.129 ± 0.085 | 0.154 ± 0.066 | 0.765 |
| Hippocampus-R | 0.176 ± 0.029 | 0.181 ± 0.024 | 0.833 |
| Amygdala-R | 0.189 ± 0.017 | 0.175 ± 0.036 | 0.765 |
| Accumbens-area-R | 0.176 ± 0.034 | 0.174 ± 0.037 | 0.937 |

All values are expressed as the area under the curve across the density range and are adjusted with age and gender. CI: Cognitive impairment; FDR: False discovery rate; L: Left; PD: Parkinson’s disease; R: Right.

Supplementary table 3. Nodal Local efficiency of PD patients with and without CI

|  | PD with CI (n = 18) | PD without CI (n = 33) | *P* value after FDR correction |
| --- | --- | --- | --- |
| Caudal anterior cingulate-L | 0.210 ± 0.012 | 0.202 ± 0.038 | 0.780 |
| Caudal middle frontal-L | 0.201 ± 0.034 | 0.207 ± 0.018 | 0.780 |
| Cuneus-L | 0.194 ± 0.041 | 0.204 ± 0.034 | 0.780 |
| Entorhinal-L | 0.181 ± 0.076 | 0.200 ± 0.041 | 0.780 |
| Fusiform-L | 0.181 ± 0.074 | 0.212 ± 0.010 | 0.780 |
| Inferior parietal-L | 0.214 ± 0.010 | 0.208 ± 0.022 | 0.780 |
| Inferior temporal-L | 0.197 ± 0.036 | 0.196 ± 0.053 | 0.993 |
| Isthmus cingulate-L | 0.214 ± 0.009 | 0.200 ± 0.039 | 0.780 |
| Lateral occipital-L | 0.208 ± 0.011 | 0.200 ± 0.041 | 0.780 |
| Lateral orbitofrontal-L | 0.195 ± 0.052 | 0.210 ± 0.018 | 0.780 |
| Lingual-L | 0.209 ± 0.009 | 0.202 ± 0.033 | 0.780 |
| Medial orbitofrontal-L | 0.196 ± 0.055 | 0.212 ± 0.008 | 0.780 |
| Middle temporal-L | 0.205 ± 0.012 | 0.207 ± 0.024 | 0.839 |
| Parahippocampal-L | 0.208 ± 0.010 | 0.197 ± 0.043 | 0.780 |
| Paracentral-L | 0.204 ± 0.036 | 0.210 ± 0.018 | 0.795 |
| Pars opercularis-L | 0.209 ± 0.006 | 0.207 ± 0.016 | 0.839 |
| Pars orbitalis-L | 0.208 ± 0.012 | 0.196 ± 0.043 | 0.780 |
| Pars triangularis-L | 0.210 ± 0.010 | 0.205 ± 0.030 | 0.791 |
| Pericalcarine-L | 0.212 ± 0.010 | 0.210 ± 0.015 | 0.839 |
| Postcentral-L | 0.210 ± 0.009 | 0.211 ± 0.016 | 0.938 |
| Posterior cingulate-L | 0.201 ± 0.048 | 0.211 ± 0.014 | 0.780 |
| Precentral-L | 0.210 ± 0.008 | 0.209 ± 0.015 | 0.938 |
| Precuneus-L | 0.201 ± 0.051 | 0.205 ± 0.032 | 0.918 |
| Rostral anterior cingulate-L | 0.209 ± 0.021 | 0.210 ± 0.015 | 0.943 |
| Rostral middle frontal-L | 0.213 ± 0.007 | 0.210 ± 0.009 | 0.780 |
| Superior frontal-L | 0.208 ± 0.011 | 0.208 ± 0.021 | 0.993 |
| Superior parietal-L | 0.213 ± 0.010 | 0.210 ± 0.013 | 0.780 |
| Superior temporal-L | 0.212 ± 0.009 | 0.204 ± 0.018 | 0.780 |
| Supramarginal-L | 0.202 ± 0.033 | 0.210 ± 0.013 | 0.780 |
| Transverse temporal-L | 0.207 ± 0.015 | 0.202 ± 0.040 | 0.839 |
| Insula-L | 0.200 ± 0.036 | 0.211 ± 0.019 | 0.780 |
| Thalamus-L | 0.199 ± 0.028 | 0.205 ± 0.035 | 0.839 |
| Caudate-L | 0.210 ± 0.014 | 0.195 ± 0.050 | 0.780 |
| Putamen-L | 0.166 ± 0.083 | 0.199 ± 0.046 | 0.780 |
| Pallidum-L | 0.170 ± 0.080 | 0.188 ± 0.059 | 0.780 |
| Hippocampus-L | 0.196 ± 0.050 | 0.203 ± 0.030 | 0.839 |
| Amygdala-L | 0.197 ± 0.045 | 0.197 ± 0.048 | 0.993 |
| Accumbens-area-L | 0.191 ± 0.062 | 0.199 ± 0.044 | 0.839 |
| Caudal anterior cingulate-R | 0.184 ± 0.068 | 0.189 ± 0.060 | 0.938 |
| Caudal middle frontal-R | 0.200 ± 0.031 | 0.212 ± 0.007 | 0.780 |
| Cuneus-R | 0.212 ± 0.007 | 0.212 ± 0.010 | 0.993 |
| Entorhinal-R | 0.197 ± 0.053 | 0.202 ± 0.028 | 0.839 |
| Fusiform-R | 0.191 ± 0.060 | 0.204 ± 0.039 | 0.780 |
| Inferior parietal-R | 0.214 ± 0.011 | 0.211 ± 0.009 | 0.780 |
| Inferior temporal-R | 0.204 ± 0.019 | 0.205 ± 0.034 | 0.990 |
| Isthmus cingulate-R | 0.210 ± 0.008 | 0.204 ± 0.042 | 0.839 |
| Lateral occipital-R | 0.192 ± 0.063 | 0.203 ± 0.039 | 0.780 |
| Lateral orbitofrontal-R | 0.210 ± 0.010 | 0.211 ± 0.011 | 0.839 |
| Lingual-R | 0.213 ± 0.009 | 0.187 ± 0.060 | 0.780 |
| Medial orbitofrontal-R | 0.194 ± 0.051 | 0.205 ± 0.039 | 0.780 |
| Middle temporal-R | 0.215 ± 0.015 | 0.203 ± 0.030 | 0.780 |
| Parahippocampal-R | 0.208 ± 0.009 | 0.203 ± 0.027 | 0.780 |
| Paracentral-R | 0.201 ± 0.052 | 0.204 ± 0.039 | 0.938 |
| Pars opercularis-R | 0.205 ± 0.021 | 0.213 ± 0.012 | 0.780 |
| Pars orbitalis-R | 0.194 ± 0.046 | 0.197 ± 0.044 | 0.938 |
| Pars triangularis-R | 0.187 ± 0.056 | 0.203 ± 0.039 | 0.780 |
| Pericalcarine-R | 0.213 ± 0.008 | 0.202 ± 0.038 | 0.780 |
| Postcentral-R | 0.196 ± 0.051 | 0.206 ± 0.039 | 0.780 |
| Posterior cingulate-R | 0.197 ± 0.050 | 0.190 ± 0.052 | 0.839 |
| Precentral-R | 0.193 ± 0.056 | 0.198 ± 0.051 | 0.881 |
| Precuneus-R | 0.210 ± 0.006 | 0.212 ± 0.012 | 0.839 |
| Rostral anterior cingulate-R | 0.208 ± 0.016 | 0.198 ± 0.052 | 0.780 |
| Rostral middle frontal-R | 0.207 ± 0.014 | 0.212 ± 0.011 | 0.780 |
| Superior frontal-R | 0.212 ± 0.007 | 0.209 ± 0.016 | 0.780 |
| Superior parietal-R | 0.212 ± 0.012 | 0.215 ± 0.009 | 0.780 |
| Superior temporal-R | 0.207 ± 0.009 | 0.210 ± 0.010 | 0.780 |
| Supramarginal-R | 0.186 ± 0.068 | 0.208 ± 0.016 | 0.780 |
| Transverse temporal-R | 0.203 ± 0.033 | 0.206 ± 0.022 | 0.881 |
| Insula-R | 0.210 ± 0.009 | 0.208 ± 0.038 | 0.938 |
| Thalamus-R | 0.195 ± 0.055 | 0.201 ± 0.041 | 0.839 |
| Caudate-R | 0.202 ± 0.026 | 0.191 ± 0.059 | 0.780 |
| Putamen-R | 0.176 ± 0.076 | 0.205 ± 0.038 | 0.780 |
| Pallidum-R | 0.148 ± 0.094 | 0.174 ± 0.073 | 0.780 |
| Hippocampus-R | 0.203 ± 0.024 | 0.207 ± 0.026 | 0.839 |
| Amygdala-R | 0.213 ± 0.014 | 0.200 ± 0.040 | 0.780 |
| Accumbens-area-R | 0.200 ± 0.037 | 0.200 ± 0.040 | 0.996 |

All values are expressed as the area under the curve across the density range and are adjusted with age and gender. CI: Cognitive impairment; FDR: False discovery rate; L: Left; PD: Parkinson’s disease; R: Right.

Supplementary table 4. Nodal degree centrality of PD patients with and without CI

|  | PD with CI (n = 18) | PD without CI (n = 33) | *P* value after FDR correction |
| --- | --- | --- | --- |
| Caudal anterior cingulate-L | 3.96 ± 1.68 | 4.15 ± 1.69 | 0.993 |
| Caudal middle frontal-L | 4.21 ± 1.28 | 4.39 ± 1.53 | 0.993 |
| Cuneus-L | 3.94 ± 1.81 | 3.98 ± 1.50 | 0.993 |
| Entorhinal-L | 3.28 ± 1.98 | 3.94 ± 1.75 | 0.769 |
| Fusiform-L | 2.88 ± 1.88 | 3.97 ± 1.34 | 0.513 |
| Inferior parietal-L | 4.78 ± 1.33 | 4.42 ± 1.49 | 0.870 |
| Inferior temporal-L | 3.10 ± 2.04 | 4.09 ± 1.64 | 0.643 |
| Isthmus cingulate-L | 4.07 ± 1.90 | 3.26 ± 1.58 | 0.691 |
| Lateral occipital-L | 4.55 ± 1.65 | 3.62 ± 1.73 | 0.643 |
| Lateral orbitofrontal-L | 3.67 ± 1.82 | 4.08 ± 1.28 | 0.810 |
| Lingual-L | 3.92 ± 1.40 | 4.15 ± 1.37 | 0.993 |
| Medial orbitofrontal-L | 4.07 ± 1.96 | 4.44 ± 1.40 | 0.899 |
| Middle temporal-L | 3.81 ± 1.76 | 4.24 ± 1.29 | 0.803 |
| Parahippocampal-L | 3.51 ± 1.19 | 3.51 ± 1.88 | 0.993 |
| Paracentral-L | 3.36 ± 1.53 | 3.91 ± 1.47 | 0.769 |
| Pars opercularis-L | 4.84 ± 1.22 | 4.04 ± 1.64 | 0.643 |
| Pars orbitalis-L | 4.40 ± 1.63 | 3.64 ± 1.83 | 0.769 |
| Pars triangularis-L | 4.66 ± 1.39 | 3.79 ± 1.34 | 0.516 |
| Pericalcarine-L | 4.03 ± 1.41 | 3.70 ± 1.61 | 0.907 |
| Postcentral-L | 4.43 ± 1.38 | 4.25 ± 1.69 | 0.993 |
| Posterior cingulate-L | 4.12 ± 1.74 | 4.00 ± 1.91 | 0.993 |
| Precentral-L | 4.74 ± 1.11 | 4.26 ± 1.50 | 0.769 |
| Precuneus-L | 4.02 ± 1.87 | 4.52 ± 1.46 | 0.769 |
| Rostral anterior cingulate-L | 4.02 ± 1.71 | 3.84 ± 1.64 | 0.993 |
| Rostral middle frontal-L | 4.48 ± 1.46 | 4.24 ± 1.41 | 0.993 |
| Superior frontal-L | 4.60 ± 1.50 | 4.41 ± 1.37 | 0.993 |
| Superior parietal-L | 3.95 ± 1.41 | 3.97 ± 1.63 | 0.993 |
| Superior temporal-L | 5.14 ± 1.17 | 4.11 ± 1.41 | 0.513 |
| Supramarginal-L | 4.14 ± 1.51 | 4.20 ± 1.44 | 0.993 |
| Transverse temporal-L | 3.88 ± 1.75 | 4.08 ± 1.71 | 0.993 |
| Insula-L | 3.78 ± 1.97 | 3.87 ± 1.64 | 0.993 |
| Thalamus-L | 3.41 ± 2.13 | 4.03 ± 1.60 | 0.769 |
| Caudate-L | 3.80 ± 1.88 | 3.71 ± 1.58 | 0.993 |
| Putamen-L | 2.74 ± 2.16 | 3.91 ± 1.53 | 0.516 |
| Pallidum-L | 1.94 ± 1.73 | 2.99 ± 1.92 | 0.643 |
| Hippocampus-L | 3.68 ± 1.82 | 3.96 ± 1.68 | 0.993 |
| Amygdala-L | 3.83 ± 1.87 | 3.85 ± 1.92 | 0.993 |
| Accumbens-area-L | 3.83 ± 1.83 | 3.61 ± 1.70 | 0.993 |
| Caudal anterior cingulate-R | 3.15 ± 1.97 | 2.95 ± 1.87 | 0.993 |
| Caudal middle frontal-R | 4.23 ± 2.10 | 4.28 ± 1.51 | 0.993 |
| Cuneus-R | 4.52 ± 1.29 | 4.08 ± 1.44 | 0.769 |
| Entorhinal-R | 3.49 ± 1.77 | 3.47 ± 1.68 | 0.993 |
| Fusiform-R | 3.98 ± 1.89 | 4.01 ± 1.68 | 0.993 |
| Inferior parietal-R | 4.16 ± 1.50 | 4.27 ± 1.38 | 0.993 |
| Inferior temporal-R | 3.85 ± 1.70 | 3.92 ± 1.90 | 0.993 |
| Isthmus cingulate-R | 4.50 ± 1.87 | 3.81 ± 1.74 | 0.769 |
| Lateral occipital-R | 4.55 ± 1.93 | 3.98 ± 1.71 | 0.769 |
| Lateral orbitofrontal-R | 4.21 ± 1.38 | 4.08 ± 1.66 | 0.993 |
| Lingual-R | 4.39 ± 1.69 | 3.69 ± 1.82 | 0.769 |
| Medial orbitofrontal-R | 3.54 ± 1.84 | 4.12 ± 1.62 | 0.769 |
| Middle temporal-R | 4.26 ± 1.67 | 4.10 ± 1.51 | 0.993 |
| Parahippocampal-R | 3.83 ± 1.54 | 3.77 ± 1.61 | 0.993 |
| Paracentral-R | 3.64 ± 1.92 | 4.05 ± 1.83 | 0.906 |
| Pars opercularis-R | 4.27 ± 1.64 | 4.03 ± 1.61 | 0.993 |
| Pars orbitalis-R | 3.61 ± 1.84 | 3.67 ± 1.86 | 0.993 |
| Pars triangularis-R | 3.18 ± 1.90 | 3.75 ± 1.57 | 0.769 |
| Pericalcarine-R | 4.39 ± 1.58 | 3.93 ± 1.68 | 0.803 |
| Postcentral-R | 4.73 ± 1.81 | 4.06 ± 1.81 | 0.769 |
| Posterior cingulate-R | 4.32 ± 1.74 | 3.81 ± 1.66 | 0.769 |
| Precentral-R | 3.98 ± 1.95 | 4.52 ± 1.52 | 0.769 |
| Precuneus-R | 4.36 ± 1.50 | 4.27 ± 1.53 | 0.993 |
| Rostral anterior cingulate-R | 3.80 ± 1.85 | 3.97 ± 1.85 | 0.993 |
| Rostral middle frontal-R | 3.80 ± 1.72 | 4.15 ± 1.27 | 0.893 |
| Superior frontal-R | 5.15 ± 1.15 | 4.62 ± 1.14 | 0.691 |
| Superior parietal-R | 3.98 ± 1.62 | 3.95 ± 1.53 | 0.993 |
| Superior temporal-R | 4.82 ± 1.29 | 4.14 ± 1.49 | 0.691 |
| Supramarginal-R | 3.67 ± 2.06 | 4.22 ± 1.62 | 0.769 |
| Transverse temporal-R | 4.01 ± 1.78 | 4.00 ± 1.56 | 0.993 |
| Insula-R | 4.30 ± 1.42 | 4.29 ± 1.72 | 0.993 |
| Thalamus-R | 2.88 ± 2.10 | 3.30 ± 1.62 | 0.899 |
| Caudate-R | 3.89 ± 1.93 | 3.88 ± 1.87 | 0.993 |
| Putamen-R | 2.83 ± 2.19 | 4.09 ± 1.43 | 0.513 |
| Pallidum-R | 2.34 ± 2.29 | 3.15 ± 1.95 | 0.769 |
| Hippocampus-R | 3.92 ± 1.47 | 3.78 ± 1.44 | 0.993 |
| Amygdala-R | 4.69 ± 1.43 | 3.87 ± 1.74 | 0.691 |
| Accumbens-area-R | 4.33 ± 1.87 | 3.94 ± 1.82 | 0.908 |

All values are adjusted with age and gender and are expressed as mean ± SD. CI: Cognitive impairment; FDR: False discovery rate; L: Left; PD: Parkinson’s disease; R: Right; SD: Standard deviation.

Supplementary table 5. Nodal betweenness centrality of PD patients with and without CI

|  | PD with CI (n = 18) | PD without CI (n = 33) | *P* value after FDR correction |
| --- | --- | --- | --- |
| Caudal anterior cingulate-L | 22.13 ± 15.61 | 23.33 ± 17.26 | 0.995 |
| Caudal middle frontal-L | 22.28 ± 10.57 | 23.86 ± 16.43 | 0.995 |
| Cuneus-L | 25.37 ± 20.29 | 20.25 ± 11.20 | 0.937 |
| Entorhinal-L | 15.74 ± 15.71 | 20.39 ± 12.79 | 0.937 |
| Fusiform-L | 14.91 ± 10.73 | 21.89 ± 15.00 | 0.937 |
| Inferior parietal-L | 19.68 ± 14.48 | 21.49 ± 11.76 | 0.995 |
| Inferior temporal-L | 21.53 ± 28.56 | 21.40 ± 12.02 | 0.995 |
| Isthmus cingulate-L | 15.07 ± 8.44 | 19.57 ± 13.14 | 0.937 |
| Lateral occipital-L | 22.16 ± 14.55 | 16.75 ± 11.88 | 0.937 |
| Lateral orbitofrontal-L | 20.41 ± 12.61 | 20.17 ± 11.44 | 0.995 |
| Lingual-L | 24.96 ± 12.13 | 21.78 ± 15.63 | 0.995 |
| Medial orbitofrontal-L | 18.27 ± 13.15 | 20.50 ± 15.63 | 0.995 |
| Middle temporal-L | 22.47 ± 13.70 | 23.32 ± 13.57 | 0.995 |
| Parahippocampal-L | 22.33 ± 13.15 | 17.85 ± 11.97 | 0.937 |
| Paracentral-L | 16.35 ± 10.51 | 17.36 ± 13.43 | 0.995 |
| Pars opercularis-L | 25.20 ± 11.83 | 19.21 ± 11.90 | 0.937 |
| Pars orbitalis-L | 25.66 ± 18.81 | 24.36 ± 24.20 | 0.995 |
| Pars triangularis-L | 18.60 ± 8.40 | 25.00 ± 22.07 | 0.937 |
| Pericalcarine-L | 18.02 ± 9.79 | 20.62 ± 13.71 | 0.995 |
| Postcentral-L | 18.47 ± 9.01 | 19.25 ± 15.47 | 0.995 |
| Posterior cingulate-L | 17.92 ± 11.70 | 17.42 ± 12.17 | 0.995 |
| Precentral-L | 20.09 ± 7.96 | 20.80 ± 14.52 | 0.995 |
| Precuneus-L | 13.96 ± 8.16 | 21.60 ± 11.19 | 0.937 |
| Rostral anterior cingulate-L | 16.32 ± 8.37 | 21.70 ± 13.94 | 0.937 |
| Rostral middle frontal-L | 19.08 ± 12.01 | 23.51 ± 18.63 | 0.995 |
| Superior frontal-L | 22.56 ± 14.14 | 23.22 ± 15.66 | 0.995 |
| Superior parietal-L | 18.34 ± 11.68 | 19.92 ± 11.79 | 0.995 |
| Superior temporal-L | 19.35 ± 7.90 | 24.32 ± 14.80 | 0.937 |
| Supramarginal-L | 20.37 ± 8.05 | 20.39 ± 13.39 | 0.995 |
| Transverse temporal-L | 18.86 ± 11.23 | 20.74 ± 14.06 | 0.995 |
| Insula-L | 15.31 ± 10.40 | 21.31 ± 16.87 | 0.937 |
| Thalamus-L | 24.19 ± 19.35 | 18.69 ± 12.50 | 0.937 |
| Caudate-L | 17.48 ± 10.41 | 25.34 ± 15.47 | 0.937 |
| Putamen-L | 21.78 ± 23.86 | 21.44 ± 17.21 | 0.995 |
| Pallidum-L | 13.62 ± 16.38 | 16.92 ± 13.91 | 0.995 |
| Hippocampus-L | 18.41 ± 12.17 | 18.82 ± 12.12 | 0.995 |
| Amygdala-L | 20.05 ± 14.61 | 22.50 ± 20.01 | 0.995 |
| Accumbens-area-L | 17.39 ± 9.01 | 18.11 ± 12.04 | 0.995 |
| Caudal anterior cingulate-R | 21.74 ± 16.29 | 15.58 ± 12.78 | 0.937 |
| Caudal middle frontal-R | 19.48 ± 11.88 | 19.64 ± 11.47 | 0.995 |
| Cuneus-R | 18.95 ± 8.47 | 21.13 ± 15.48 | 0.995 |
| Entorhinal-R | 18.47 ± 14.21 | 18.64 ± 13.36 | 0.995 |
| Fusiform-R | 17.81 ± 11.37 | 23.30 ± 14.38 | 0.937 |
| Inferior parietal-R | 17.88 ± 13.06 | 17.79 ± 8.57 | 0.995 |
| Inferior temporal-R | 23.95 ± 18.47 | 19.05 ± 18.30 | 0.995 |
| Isthmus cingulate-R | 20.24 ± 12.93 | 18.53 ± 13.57 | 0.995 |
| Lateral occipital-R | 21.01 ± 15.92 | 19.37 ± 12.61 | 0.995 |
| Lateral orbitofrontal-R | 21.37 ± 10.92 | 20.50 ± 15.53 | 0.995 |
| Lingual-R | 18.43 ± 10.19 | 20.85 ± 21.02 | 0.995 |
| Medial orbitofrontal-R | 19.75 ± 12.93 | 19.37 ± 14.00 | 0.995 |
| Middle temporal-R | 18.08 ± 27.70 | 22.48 ± 15.47 | 0.995 |
| Parahippocampal-R | 22.55 ± 11.22 | 24.18 ± 16.22 | 0.995 |
| Paracentral-R | 13.00 ± 8.19 | 20.53 ± 15.89 | 0.937 |
| Pars opercularis-R | 21.35 ± 15.15 | 17.49 ± 10.65 | 0.960 |
| Pars orbitalis-R | 24.56 ± 21.16 | 22.74 ± 16.80 | 0.995 |
| Pars triangularis-R | 21.61 ± 14.13 | 19.22 ± 15.84 | 0.995 |
| Pericalcarine-R | 17.25 ± 9.71 | 19.82 ± 16.01 | 0.995 |
| Postcentral-R | 19.91 ± 10.58 | 19.00 ± 13.78 | 0.995 |
| Posterior cingulate-R | 23.22 ± 14.86 | 24.73 ± 17.13 | 0.995 |
| Precentral-R | 22.33 ± 17.24 | 23.38 ± 16.89 | 0.995 |
| Precuneus-R | 22.23 ± 11.96 | 19.02 ± 11.92 | 0.995 |
| Rostral anterior cingulate-R | 15.31 ± 8.77 | 17.49 ± 11.66 | 0.995 |
| Rostral middle frontal-R | 22.43 ± 18.25 | 19.99 ± 12.25 | 0.995 |
| Superior frontal-R | 22.25 ± 13.82 | 21.93 ± 12.00 | 0.995 |
| Superior parietal-R | 22.75 ± 19.30 | 17.54 ± 9.31 | 0.937 |
| Superior temporal-R | 28.57 ± 17.49 | 22.36 ± 10.86 | 0.937 |
| Supramarginal-R | 16.76 ± 12.52 | 19.75 ± 12.49 | 0.995 |
| Transverse temporal-R | 23.34 ± 20.65 | 23.92 ± 16.93 | 0.995 |
| Insula-R | 21.48 ± 16.12 | 17.47 ± 11.25 | 0.960 |
| Thalamus-R | 18.46 ± 33.42 | 17.38 ± 11.25 | 0.995 |
| Caudate-R | 22.95 ± 15.65 | 18.58 ± 12.44 | 0.960 |
| Putamen-R | 12.43 ± 10.41 | 17.18 ± 12.38 | 0.937 |
| Pallidum-R | 13.04 ± 15.67 | 14.64 ± 10.51 | 0.995 |
| Hippocampus-R | 24.76 ± 11.79 | 20.16 ± 9.84 | 0.937 |
| Amygdala-R | 17.95 ± 7.35 | 17.91 ± 10.27 | 0.995 |
| Accumbens-area-R | 18.67 ± 9.85 | 20.08 ± 14.07 | 0.995 |

All values are adjusted with age and gender and are expressed as mean ± SD. CI: Cognitive impairment; FDR: False discovery rate; L: Left; PD: Parkinson’s disease; R: Right; SD: Standard deviation.
